# Comparative sensitivity to methyl eugenol of four putative *Bactrocera dorsalis* complex sibling species – further evidence that they belong to one and the same species *B. dorsalis*

**DOI:** 10.3897/zookeys.540.6099

**Published:** 2015-11-26

**Authors:** Alvin K.W. Hee, Yue-Shin Ooi, Suk-Ling Wee, Keng-Hong Tan

**Affiliations:** 1 Department of Biology, Faculty of Science, Universiti Putra Malaysia, 43400 UPM Serdang, Selangor Darul Ehsan, Malaysia; 2 School of Environmental and Natural Resource Sciences; 3 Centre of Insect Systematics, Faculty of Science and Technology, Universiti Kebangsaan Malaysia, Bangi, Malaysia; 4 Tan Hak Heng Co., Penang, Malaysia

**Keywords:** *Bactrocera dorsalis*, *Bactrocera invadens*, *Bactrocera papayae*, *Bactrocera philippinensis*, methyl eugenol, male response, lure sensitivity

## Abstract

Males of certain species belonging to the *Bactrocera dorsalis* complex are strongly attracted to, and readily feed on methyl eugenol (ME), a plant secondary compound that is found in over 480 plant species worldwide. Amongst those species is one of the world’s most severe fruit pests the Oriental fruit fly, *Bactrocera dorsalis* *s.s.*, and the former taxonomic species *Bactrocera invadens*, *Bactrocera papayae* and *Bactrocera philippinensis*. The latter species have been recently synonymised with *Bactrocera dorsalis* based on their very similar morphology, mating compatibility, molecular genetics and identical sex pheromones following consumption of ME. Previous studies have shown that male fruit fly responsiveness to lures is a unique phenomenon that is dose species-specific, besides showing a close correlation to sexual maturity attainment. This led us to use ME sensitivity as a behavioural parameter to test if *Bactrocera dorsalis* and the three former taxonomic species had similar sensitivity towards odours of ME. Using Probit analysis, we estimated the median dose of ME required to elicit species’ positive response in 50% of each population tested (ED_50_). ED_50 _values were compared between *Bactrocera dorsalis* and the former species. Our results showed no significant differences between *Bactrocera dorsalis* *s.s.*, and the former *Bactrocera invadens*, *Bactrocera papayae* and *Bactrocera philippinensis* in their response to ME. We consider that the *Bactrocera* males’ sensitivity to ME may be a useful behavioural parameter for species delimitation and, in addition to other integrative taxonomic tools used, provides further supportive evidence that the four taxa belong to one and the same biological species, *Bactrocera dorsalis*.

## Introduction

A number of fruit fly species in the *Bactrocera dorsalis* complex are pests of economic importance. The most notorious is the Oriental fruit fly, *Bactrocera dorsalis* (Hendel), a widely distributed and invasive species which includes the recently synonymised *Bactrocera invadens* Drew, Tsuruta & White, *Bactrocera papayae* Drew & Hancock and *Bactrocera philippinensis* Drew & Hancock. The presence of this species in the tropics and subtropics has caused significant damage by rendering infested fruits inedible and prohibiting fruit exports due to strict quarantine restrictions. Direct and indirect losses attributed to pestiferous tephritids are believed to be over US$2 billion annually (Malavasi 2014). As a case in point, the 2014 ban on the entry of the popular mango (*Mangifera indica*) cv. ‘Alphonso’ from India to the EU countries (European Commission 2014) due to detection of Oriental fruit fly, made headlines globally and created an uproar in India, which is the largest mango producer in the world. Although the ban was lifted in January 2015 (European Commission 2015), the duration of the ban had adversely affected the lucrative mango export and livelihood of growers.

Further adding to the global fruit fly problem was the incursion into Africa of suspected Oriental fruit fly in 2003 (Lux et al. 2003), subsequently described as a new species, *Bactrocera invadens*, two years later (Drew et al. 2005). The *Bactrocera invadens’* African invasion was so serious that within three years of first incursion into Kenya, it had already been found to attack over 50 types of fruits in over 20 countries (Ekesi et al. 2006). Whilst *Bactrocera invadens*, *Bactrocera dorsalis*, *Bactrocera papayae* and *Bactrocera philippinensis* were known to appear similar morphologically and genetically, could interbreed and produce viable offspring as well as having identical male sex pheromone components (Tan et al. 2011, 2013, Schutze et al. 2013), it was only recently that all four species were synonymized as *Bactrocera dorsalis* (Schutze et al. 2015).

Previous studies have shown that male fruit fly responsiveness to the male lure methyl eugenol (ME) is a unique phenomenon that is dose species-specific, besides showing a close correlation to sexual maturity attainment (Wong et al. 1989, 1991, Wee and Tan 2000a). For example, *Bactrocera papayae* and *Bactrocera dorsalis* demonstrate similar ranges of sensitivity to ME at nanogramme levels, while *Bactrocera carambolae* has been shown to be at least 10 times less sensitive to ME compared to the former two species (Wee et al 2002). This led us to hypothesize that if *Bactrocera dorsalis*, *Bactrocera invadens*, *Bactrocera philippinensis* and *Bactrocera papayae* are different names for the one biological species, then their sensitivity to ME could be used as a behavioural parameter to confirm, or refute that assumption, i.e. we evaluated if the sensitivity of those putative species to ME were significantly different (as might be expected if different biological species), or were the same (as might be expected if they are populations of the same biological species). In this paper, the minimum dose of ME needed to elicit species’ positive response in 50% of the population tested (ED_50_) was determined using Probit analysis and compared between the putative species as a species’ delimiting tool.

## Methods

### Insects

Colonies of adult *Bactrocera dorsalis* and the former *Bactrocera invadens*, *Bactrocera philippinensis* and *Bactrocera papayae* were maintained in UPM insectary under strict quarantine. *Bactrocera papayae* were raised from locally collected infested starfruits, *Averrhoa carambola* L.; while *Bactrocera dorsalis*, *Bactrocera invadens*, *Bactrocera philippinensis* were obtained as pupae from the FAO/IAEA Insect Pest Control Laboratory in Seibersdorf, Austria in 2010. Pupae were imported into Malaysia using permits issued by the Director-General of Department of Agriculture Malaysia to AKW Hee. The origins of the Seibersdorf cultures are as follows: *Bactrocera dorsalis*, Saraburi, Thailand; *Bactrocera invadens*, Kenya; and *Bactrocera philippinensis*, Guimaras, the Philippines. All adult flies were provided with water and a mixture of sugar and hydrolysed protein (3:1 w/w) *ad libitum*. The flies were bred under conditions of 25–29 °C with 83–90% relative humidity, and a 12 L: 12 D photoperiod. Male flies were separated within three days of emergence (DAE) to prevent mating and were maintained in separate cages (30 cm × 30 cm × 30 cm) until required for bioassay at 19 DAE.

### Chemical preparation

Different concentrations of ME (50, 100, 300, 500, 700 and 1000 ng per 5 µl of absolute ethanol, respectively) were prepared by serial dilution from pure ME (>99.8% purity; Merck-Schuchardt, Germany) following preliminary dose-response tests that showed attraction of male flies to ME of between 15–85%, which is in the linear portion of the population response curve (Heong et al. 2013). The use of six dilutions exceeds the minimum of five suggested for studies of this type by Robertson et al. (2007).

### Probit regression of male flies’ attraction to methyl eugenol

Laboratory bioassays with sexually mature and virgin male flies for their attraction to ME were conducted with slight modifications from the protocol of Wee et al. (2002). Males (30 males per replicate per cage [40 cm × 40 cm × 40 cm]) were acclimatized for 24 h before experimentation, in an isolated indoor area to prevent exposure of ME to other colonies of flies that were yet to be assayed for ME response. Starting with 50 ng, the lowest concentration, 5 µl of diluted ME was dispensed using a Hamilton® 5 µl syringe onto a filter paper disc (3 cm × 3cm; Whatman® No.1) placed in a disposable Petri dish. During the bioassay, we promptly removed any male that approached the ME spot and attempted to feed. After 5 min, the total number of flies that responded positively was recorded. The same procedure was conducted for new batches of males at higher ME concentrations. There were between 5–9 replicates performed for each dosage and putative species using flies from different cohorts on different days. Absolute ethanol (>99%) was used as a control. All used filter papers and petri dishes were securely disposed in airtight containers to remove traces of ME from the laboratory environment.

The data obtained were pooled and subjected to Probit analyses using the PoloPlus software (LeOra Software 2002) based on Finney (1971). Probit regressions along with ED_50 _(effective median dose – that dose which elicits a response in 50% of the population tested) values were generated. Regression lines fitted to the dose response curves were subjected to a parallelism test using PoloPlus. This is necessary as relative potency, a measure of the species’ responses to ME (based on ratios of ED_50_), can only be valid when those ME dose-response regression lines are found to be parallel (Robertson et al. 2007; Heong et al. 2013).

## Results

**Figure 1.**
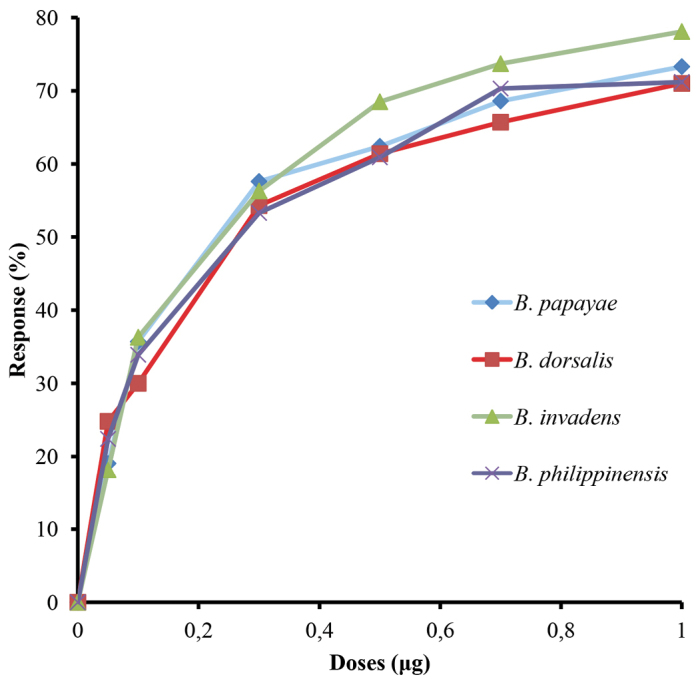
Dose-sensitivity response curves of *Bactrocera dorsalis* and the former taxa *Bactrocera papayae*, *Bactrocera invadens* and *Bactrocera philippinensis* to methyl eugenol at different doses.

**Figure 2.**
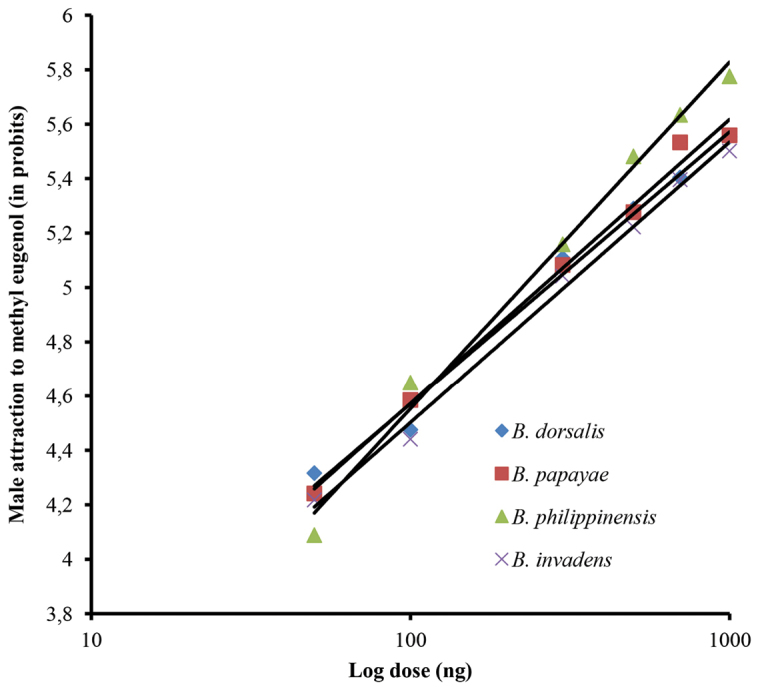
Probit lines of *Bactrocera dorsalis* and the former taxa *Bactrocera papayae*, *Bactrocera philippinensis* and *Bactrocera invadens* attraction to methyl eugenol.

The male flies displayed typical behaviour in response to ME i.e. immediate zig-zag flying in locating the source of ME upon sensing the lure, followed by compulsive feeding on ME. When offered to the flies, ME attracted all four putative species at all of the tested doses (Figure 1). Approximately 25% of *Bactrocera dorsalis* responded to 50 ng of ME, which was the lowest concentration of ME tested and their responses were observed to gradually increase when subjected to higher dosages (Figure 1). The ED_50_ response to ME for *Bactrocera dorsalis* males was 268 ng (Table 1). Similar behavioural responses were also observed in the males of the other three putative species. At the lowest dose of 50 ng, ME attracted 18%, 22% and 16% of *Bactrocera invadens*, *Bactrocera philippinensis* and *Bactrocera papayae* males, respectively (Figure 1), with their corresponding ED_50 _values being 222 ng, 256 ng and 247 ng (Table 1). The validity of the ED_50 _values was confirmed with heterogeneity factors of each species’ response to ME showing values < 1 (*Bactrocera dorsalis*, 0.484; *Bactrocera invadens*, 0.714; *Bactrocera philippinensis*, 0.502 and *Bactrocera papayae*, 0.859), indicating the data fits well with the model of standardized residuals when plotted against the predicted values. The likelihood ratio test of parallelism revealed that the slopes of the regression lines for *Bactrocera dorsalis*, *Bactrocera invadens*, *Bactrocera philippinensis* and *Bactrocera papayae* were not significantly different (Figure 2) (*p* > 0.05; λ^2^ = 7.27; df = 3). This allowed us to compare the relative potencies of the four species to ME. The attraction of *Bactrocera dorsalis*, *Bactrocera invadens*, *Bactrocera philippinensis* and *Bactrocera papayae* males to ME were found to be not significantly different based on their relative potency values which ranged between 0.9 and 1.1(Table 1). In all controls, no males were attracted to filter paper containing absolute ethanol.

**Table 1.** Probit analysis of male attractancy to methyl eugenol for *Bactrocera dorsalis* and the former taxa *Bactrocera papayae*, *Bactrocera philippinensis* and *Bactrocera invadens*.

Species	*n*^a^	Regression equation	*χ^2^*	*df*	ED_50_*^b^* (ng)	95% fiducial limits (ng)		Relative potency
						Lower	Upper	
*Bactrocera papayae*	210	y=1.108x-2.651	3.435	4	247	211	287	1.0
*Bactrocera philippinensis*	330	y=1.043x-2.511	2.008	4	256	224	290	1.0
*Bactrocera invadens*	270	y=1.266x-2.970	2.857	4	222	195	250	0.9
*Bactrocera dorsalis*	210	y=1.002x-2.433	1.936	4	268	226	316	1.1

a *n* - number of insects tested per dose

b ED_50 _(effective median dose) is the dose required to elicit a positive response in 50% of the fruit fly population tested.

## Discussion

*Bactrocera dorsalis* and the three former species *Bactrocera invadens*, *Bactrocera philippinensis* and *Bactrocera papayae* all showed similar sensitivity to the male lure, ME, with non-significant differences in ED_50 _and potency values. Only marginal difference in the ED_50 _between *Bactrocera papayae* and *Bactrocera dorsalis* in the current study is in contrast to our earlier work that showed approximately twice the level of ED_50 _of *Bactrocera papayae* over *Bactrocera dorsalis* (Wee et al. 2002). We believe that the discrepancy in those ratios is attributed to the different geographical strains of *Bactrocera dorsalis* used, as the strain that was used by Wee et al. originated from Taichung, Taiwan, whilst the current strain used in this study originated from Saraburi, Thailand. Further, it must be noted that there was also no significant difference in the consumption of ME between *Bactrocera dorsalis* and *Bactrocera papayae* (Wee et al. 2002). The absence of parallelism test in Wee et al. was due to the fact that the inclusion of *Bactrocera carambolae* in that study elicited a very high ED_50_ value (17 and 9 times higher than that of *Bactrocera dorsalis* and *Bactrocera papayae*) that resulted in the unsuitability of parallelism test of the regression lines for that study.

ME is found naturally in over 480 plant species (Tan and Nishida 2012) and is a pheromone precursor/booster for ME-sensitive *Bactrocera* species (Tan and Nishida 1996, Tan et al. 2014). Synthetic ME is widely used to control pestiferous *Bactrocera* species such as *Bactrocera dorsalis* in male annihilation programmes (Steiner et al. 1965, Vargas et al. 2014). However, it was not until the discovery that the responses of male fruit fly species such as *Bactrocera dorsalis* and *Bactrocera carambolae* to ME was quantifiable (Wee et al 2002), that we have used male ME response as a species’ delimitation tool. As an example, the fact that *Bactrocera carambolae* is able to interbreed with *Bactrocera papayae* and produce viable offspring both in the laboratory and in the wild (Wee and Tan 2000b, 2005, Tan 2003; but see Schutze et al. 2013) suggested that the former might be a sub-species belonging to a single species. However, that male *Bactrocera carambolae* possess significantly lower sensitivity to ME compared with that of *Bactrocera dorsalis* in cage assays (Wee et al. 2002) further prompted efforts to evaluate the status of *Bactrocera carambolae* leading to the recognition that *Bactrocera carambolae* is currently a distinct, perhaps an incipient, species from that of *Bactrocera dorsalis* (Schutze et al. 2015).

An important applied finding of this study, separate to the species delimitation issue, involves the use of ME in the field as a pest control. When ME is applied in the field, the different levels of male attraction to ME between species may impact on chances of male annihilation success. In the case of *Bactrocera dorsalis* and the former taxonomic species *Bactrocera invadens*, *Bactrocera papayae* and *Bactrocera philippinensis*, application of male annihilation technique against those flies is not expected to affect the success of the programme given that all four species (now synonymised as *Bactrocera dorsalis*) have been proven to have similar sensitivities to ME.
